# Interplay between Intravitreal RvD1 and Local Endogenous Sirtuin-1 in the Protection from Endotoxin-Induced Uveitis in Rats

**DOI:** 10.1155/2015/126408

**Published:** 2015-06-09

**Authors:** S. Rossi, C. Di Filippo, C. Gesualdo, F. Testa, M. C. Trotta, R. Maisto, B. Ferraro, F. Ferraraccio, M. Accardo, F. Simonelli, M. D'Amico

**Affiliations:** ^1^Department of Ophthalmology, Second University of Naples, 80131 Naples, Italy; ^2^Department of Experimental Medicine, Second University of Naples, 80138 Naples, Italy; ^3^Department of Clinical, Public and Preventive Medicine, Second University of Naples, 80138 Naples, Italy

## Abstract

Rat endotoxin-induced uveitis (EIU) is a well-established model of human uveitis. In this model, intravitreal injection of resolvin D1 (RvD1, 10–100–1000 ng/kg) 1 hour after subcutaneous treatment of Sprague-Dawley rats with lipopolysaccharide (LPS, 200 *μ*g/rat) significantly prevented the development of uveitis into the eye. RvD1 dose-dependently increased the expression of sirtuin-1 (SIRT1) within the eye, while it decreased the expression of acetyl-p53 and acetyl-FOXO1. These effects were accompanied by local downregulation of some microRNAs related to the expression and activity of SIRT1. These were miR-195-5p, miR-200a-3p, miR-34a-5p, and miR-145-5p. An increase of manganese superoxide dismutase and decrease of caspase 3 were evident after RvD1 treatment. In another set of experiments, the protective effects of RvD1 (1000 ng/kg) were partly abolished by the pretreatment of the rats with EX527 (10 mg/kg/day, i.p.), a specific inhibitor of SIRT1 activity, for 7 days prior to the induction of EIU in rats. Similarly, the effects of RvD1 (1000 ng/kg) on the SIRT1 protein expression were abolished by Boc2, *N*-*t*-butoxycarbonyl-PLPLP, a specific formyl-peptide receptor type 2/lipoxin A receptor antagonist. Therefore, an interplay of the SIRT1 activity on the RvD1 mediated resolution of EIU is argued.

## 1. Introduction

Endotoxin-induced uveitis (EIU) in rats is an experimental model of human uveitis. In this model, uveitis is induced by systemic injection of LPS, develops after 24 h, and has involvement of both eyes with acute anterior inflammation, disruption of the blood-ocular barrier, and accumulation of cellular inflammatory mediators [1]. Some years ago, it was found that sirtuin-1 (SIRT1), one of the most important classes of proteins with NAD^+^-dependent histone deacetylase activity [2, 3], has beneficial actions on the EIU [4–8] mainly through modification of the expression of some important miRNAs that regulate inflammatory and immune responses in the eye [9–11]. More recently, studies from our group [12] have shown that exogenous resolvin D1 (RvD1), a lipid derived protein that promotes the resolution of the inflammatory response back to a noninflamed state [13–16], reduces uveitis by improving the immune-inflammatory profile of the external and median tunics of the eye and by stimulating the resolution of the inflammation. Bearing in mind this evidence, the aim of the present study was to investigate whether there is interplay between the protein RvD1 and the protein SIRT1 in the eye protection against EIU. For this, the effects of a specific inhibitor of SIRT1 activity and a specific RvD1 receptor antagonist were, respectively, investigated on the RvD1 response and on the local expression of SIRT1.

## 2. Materials and Methods

Endotoxin-induced uveitis (EIU) was induced in rats as described by Rossi et al. [12] with some modification. Briefly, 200 *μ*g of lipopolysaccharide (LPS,* Salmonella minnesota*, Sigma, St. Louis, MO, USA) was injected subcutaneously under the paw in 0.1 mL of saline for the induction of EIU. All the surgical interventions and animal care procedures, which are in compliance with the ARVO statement for the use of Animals in Ophthalmic and Vision Research, were approved by the ethic committee at the Second University of Naples. The rats were anaesthetized by intraperitoneal injection of pentobarbital (45 mg/kg in saline) and RvD1 (10–100–1000 ng/kg) was administered intravitreously as described previously with some modifications [17–19]. One drop instillation of tropicamide 5% was used to dilate pupils while one drop of tetracaine 1% was administered for local anaesthesia. The intravitreal injection of RvD1 was performed in the right eye 1 h following LPS injection, using sterile syringes fitted with a 30-gauge needle; rats received a single intravitreal injection of 5 *µ*L [17, 18, 20] of RvD1 solution. The experimental groups (*n* = 6 rats for each group) were vehicle (saline + ethanol), saline + LPS, and LPS + RvD1 at the doses of 10–100–1000 ng/kg. In addition, in order to ascertain the pharmacological modulation of SIRT1 by RvD1, the following experimental groups (*n* = 6 rats for each group) were considered: (i) EX527 (10 mg/kg/day), a SIRT1 activity inhibitor, was intraperitoneally injected daily for 7 days [21] prior to induction of uveitis in rats (*n* = 6) receiving also RvD1 (1000 ng/kg, 1 hour following LPS injection); (ii) in the second set, LPS-treated rats (*n* = 6) were injected into the vitreous with the RvD1 FPR2/ALX receptor antagonist Boc2 (0.4 mg/kg/4 *µ*L) [22] 30 min prior to the intravitreal injection of highest doses of RvD1 1000 ng/kg. In all experimental groups, rats (*n* = 6 for each group) were killed 24 h after LPS treatment.

### 2.1. Clinical Score Attributed to EIU

After the injection of vehicle or LPS, the rats had 1 hour later intravitreal injection of RvD1. The assessment of EIU was determined as previously reported by Rossi et al. [12], scoring it from 0 to 4. Briefly, grade 0 meant no inflammation, grade 1 minimal iris and conjunctival vasodilation, grade 2 moderate iris and conjunctival vessel dilation but without evident cells in the anterior chamber, grade 3 intense iris vessels dilation and hyperemia in the anterior chamber, and grade 4 intense inflammatory reaction. EIU was considered positive when the score assigned was >1. Moreover, the tissue damage was verified by hematoxylin and eosin staining of eye sections.

### 2.2. Eye Samples

After 24 h of EIU, the eyes were harvested and cut in two halves. One half was immediately frozen in liquid nitrogen and stored at −80°C for the later biochemical assays described below, and the other was paraffin-embedded for immunohistochemistry.

### 2.3. Western Blotting Technique

Western blot technique has evaluated the expression of the following proteins: SIRT1, p53, and FOXO1. For this, frozen samples were homogenized in a solution containing 0.5% hexadecyl-trimethyl-ammonium bromide dissolved in 10 mM potassium phosphate buffer (pH 7) and centrifuged for 30 min at 4,000 ×g at 4°C. Protein sample concentration was calculated according to Bradford's method, and 15 *μ*g protein sample was used for the gel electrophoresis in a 6% PAGE separation gel. The samples were electrotransferred onto a PVDF membrane. Blots were blocked with 5% nonfat dry milk for 1 h at room temperature and then incubated with primary specific antibodies overnight, followed by incubation with a horseradish peroxidase-conjugated secondary antibody for 1 h at room temperature. Western blotting signal was visualized with an enhanced chemiluminescence detection reagent quantized by densitometry and results were expressed as densitometric units (DU). The following primary antibodies were used: anti-SIRT1 antibody (ab1103; purchased by Abcam Cambridge; UK) and anti-acetyl-p53 and anti-acetyl-FOXO1 (purchased by Santa Cruz, USA). For all assays, a secondary HRP horseradish peroxidase goat anti-mouse antibody was used (purchased by Santa Cruz, USA).

### 2.4. Immunohistochemistry

Paraffin-embedded eye samples were treated as described by Rossi et al. [12]. Eye sections were incubated with primary anti-acetyl-p53 and anti-acetyl-FOXO1 antibodies (Santa Cruz, USA) and with secondary goat anti-rabbit IgG (DBA, Milan, Italy). A negative control was performed with the primary antibody omitted (data not shown). Seven distinct tissue sections for each group of animals were done and 20 microscopic fields were analyzed in each section at 200x magnification, by an expert pathologist (intraobserver variability 6%) blinded to the experimental protocol. Of each total area, a computer-aided planimetry (IM500, Leica Microsystem, Milano, Italy) was performed and the percentage of positive stained area per total area analyzed was calculated. A color threshold mask for immunostaining was defined and applied to all sections.

### 2.5. Isolation of Total RNA from Ocular Tissue and miRNA Study

The samples were thawed and placed in dry ice. An appropriate volume of PBS (phosphate-buffer saline) was added in order to remove any impurities or residues, and an appropriate volume of QIAzol Lysis Reagent was added to perform the tissues homogenization, using the Potter homogenizer. The isolation of total RNA, including small RNAs, was performed following the MiRNeasy Minikit (Qiagen), according to the manufacturer's protocol. Syn-cel-miR-39 miScript miRNA Mimic 5 nM, added to each sample before the extraction, was used to monitor the efficiency of miRNA extraction. Total RNA was purified from 200 *µ*L of tissutal lysate and the RNA elution was performed with RNase-free water. 260/280 ratio evaluated the quality and quantity of the RNA, using NanoDrop spectrophotometer. MiScript II Reverse Transcription Kit (Qiagen, Italy) was used to convert mature miRNAs in cDNA, according to the manufacturer's protocol. The real-time PCR analysis was performed on a MyiQ2 thermocycler (Bio-Rad) using the Inflammatory Response & Autoimmunity miRNA PCR Array (MIRN-105Z). cDNA obtained from the reverse transcription reaction was added to the miScript Universal Primer (reverse primer) and QuantiTect SYBR Green PCR Master Mix (miScript SYBR Green Kit) and then to the miRNA PCR array, containing miRNA-specific miScript Primer Assays.

### 2.6. Statistical Analysis

Data analysis was performed with the web-based software package http://pcrdataanalysis.sabiosciences.com/mirna/arrayanalysis.php for the miRNA PCR array system. The ΔΔCT-method of relative quantification was used in order to obtain the expression of miRNAs in each treatment and then to compare the different profiles (range of differential expression = 1.5). Syn-mir-39, a synthetic miRNA, was used for normalization of qRT-PCR results. ΔCt value for each miRNA profiled in a plate is calculated using the formula ΔCt = Ct^miRNA^ − Ct^cel-syn-mir-39^ . ΔΔCt for each miRNA across 2 groups of samples is calculated using the formula ΔΔCt = ΔCt of treatment group −  ΔCt of control group. The fold-change for the miRNas expression was then obtained as 2^−DDCt^ (the normalized gene expression (2^−ΔCt^) in the treatment group divided by the normalized gene expression (2^−ΔCt^) in the control group). Statistical analysis was assessed either by Student's *t*-test (when only two groups were compared) or by one-way ANOVA followed by Dunnett's test (more than two experimental groups). The limit for statistical significance was expressed as *P* < 0.05.

## 3. Results

### 3.1. The Inhibition of the SIRT1 Activity Decreases the Eye Protection of RvD1

**Figure 1:**
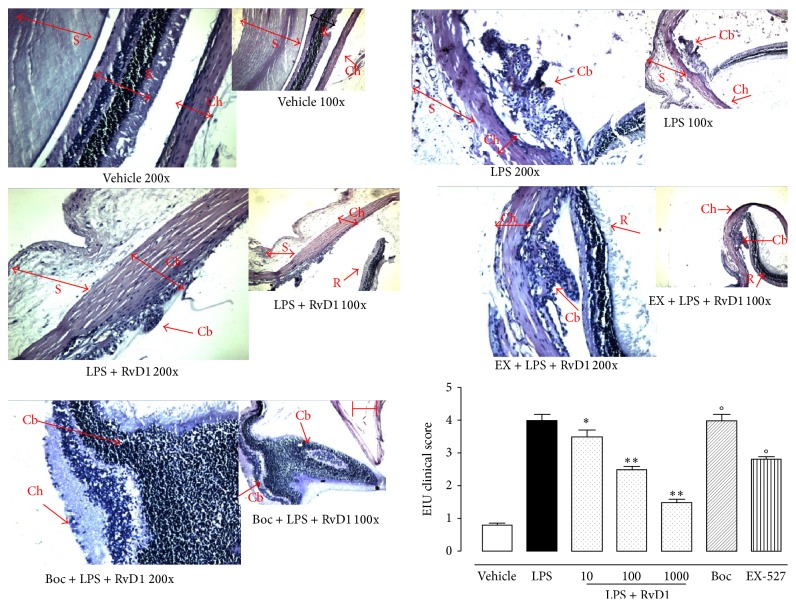
Resolvin 1 and sirtuin-1 in clinical development of EIU. Vehicle (saline + ethanol) and resolvin 1 (RvD1, 10–100–1000 ng/kg) were injected into the vitreous of rats 1 hour after vehicle + LPS (200 *µ*g/rat) treatment. Clinical manifestations of EIU were evaluated 24 hours later both with hematoxylin and with eosin (representative pictures of RvD1 1000 ng; magnification: 100x and 200x, scale bar = 100 *µ*m) and scored as reported in text (see Section 2). This figure also shows the effects of pretreatment with compound EX-527, an inhibitor of sirtuin-1 activity (10 mg/kg/day, i.p. for 7 days), prior to LPS and LPS + RvD1 (1000 ng/kg), and the effects of treatment with RvD1 FPR2/ALX Boc2 receptor antagonist (0.4 mg/kg/4 *μ*L) into the vitreous (30 min prior to RvD1 1000 ng/kg). Values are reported as the mean ± S.E.M.; *n* = 6 observations for each group. ^*∗*^ *P* < 0.05 and ^*∗∗*^ *P* < 0.01 versus LPS-treated rats; °*P* < 0.01 versus RvD1 1000 ng/kg.

Intravitreal injection of RvD1 (10, 100, 1000 ng/kg) 1 hour after LPS dose-dependently decreases the tissue damage and the clinical score attributed to EIU. This effect started from the lowest dose of RvD1 (10 ng/kg) (Figure 1, *P* < 0.05 versus LPS-treated rats) and was more evident with the two other doses (Figure 1). Interestingly, EX-527 treatment (10 mg/kg, i.p. injected daily for 7 days) prior to LPS + RvD1 significantly reduced the fall in clinical score induced by RvD1 (*P* < 0.01 versus LPS + RvD1 1000 ng/kg, Figure 1). Similarly, the treatment of the rats with the RvD1 receptor antagonist Boc2 injected into the eyes before LPS + RvD1 (1000 ng/kg) completely abolished the protection of RVD1 (Figure 1).

### 3.2. Intravitreal RvD1 and SIRT1/p53/FOXO1 Pathway

**Figure 2:**
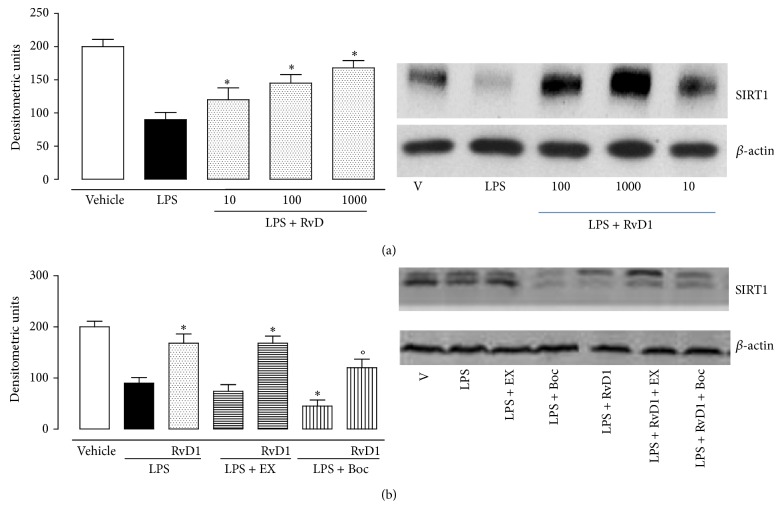
Local expression of SIRT1 following intravitreal resolvin D1 (RvD1). (a) SIRT1 expression evaluated by western blotting in the eyes of vehicle, LPS-treated rats, and LPS + RvD1 (10–100–1000 ng/kg) treated rats. (b) SIRT1 expression after EX-527, inhibitor of sirtuin-1 activity, and Boc2, FPR2/ALX receptor antagonist, in LPS and LPS + RvD1 (1000 ng/kg) rats. Results are expressed as densitometric units and represent the mean ± S.E.M. of 6 observations for each group. Significant differences with the LPS group are shown as ^*∗*^ *P* < 0.05 and ^*∗∗*^ *P* < 0.01, while differences versus LPS + RvD1 1000 ng/kg groups are shown as °*P* < 0.01.

**Figure 3:**
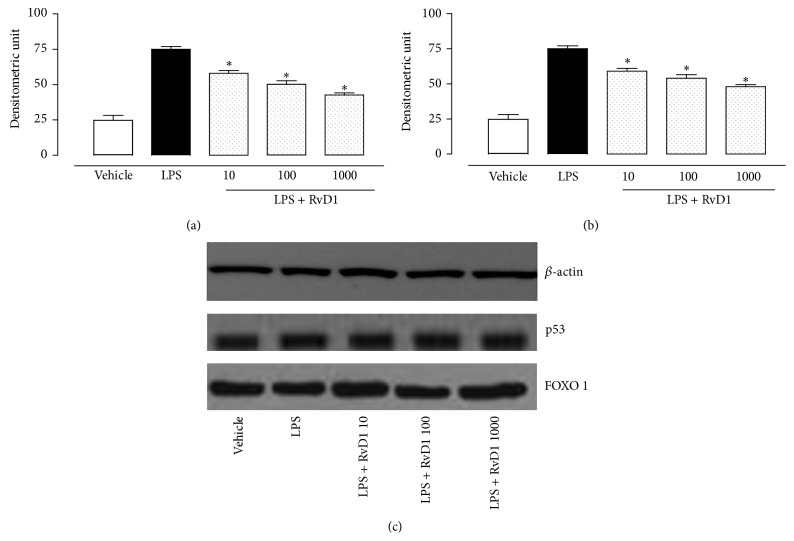
Intravitreal RvD1 (10, 100, 1000 ng/kg) in LPS rats reduced the expression of total p53 (a) and total FOXO1 (b). Values are reported as the mean ± S.E.M. of *n* = 6 observations per group. ^*∗*^ *P* < 0.01 versus LPS-treated group. RvD1 = resolvin D1.

**Figure 4:**
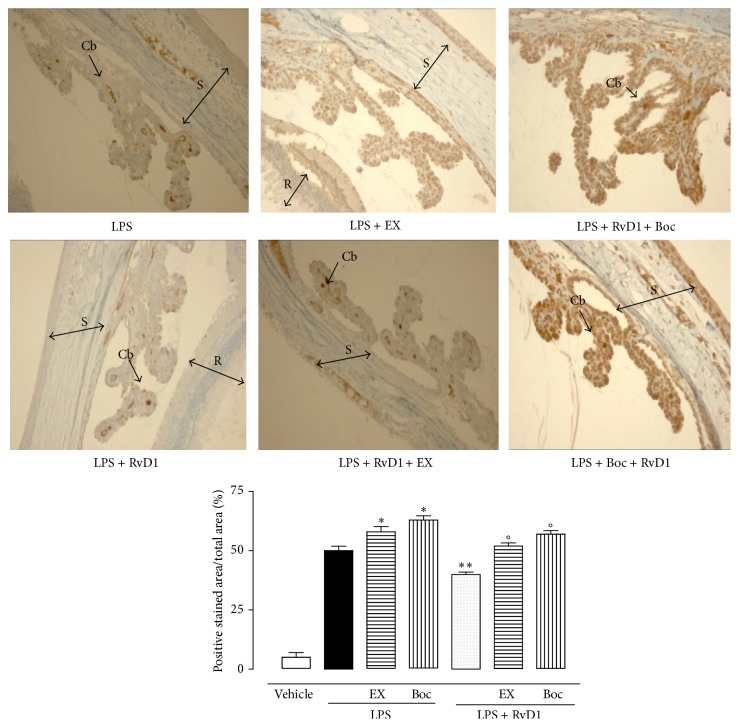
Representative immunohistochemistries of ocular tissues showing p53 in LPS rats alone; LPS + RvD1 (1000 ng/kg); EX-527 + LPS + RvD1 (1000 ng/kg); Boc2 + LPS + RvD1 (1000 ng/kg). RvD1 decreased p53 expression, as shown by the percentage of stained area per total area analyzed. The addition of EX-527 and Boc2 increases the expression of p53 with respect to LPS alone, while they decrease the expression of this factor if administered prior to LPS + RvD1 (1000 ng/kg). Values are mean ± S.E.M. of *n* = 6 observations for each group. ^*∗*^ *P* < 0.05 and ^*∗∗*^ *P* < 0.01 versus LPS-treated group, while differences versus LPS + RvD1 1000 ng/kg groups are shown as °*P* < 0.01. CB = ciliary body; R = retina; S = sclera; EX = Ex527; Boc = Boc2; RvD1 = resolvin D1.

**Figure 5:**
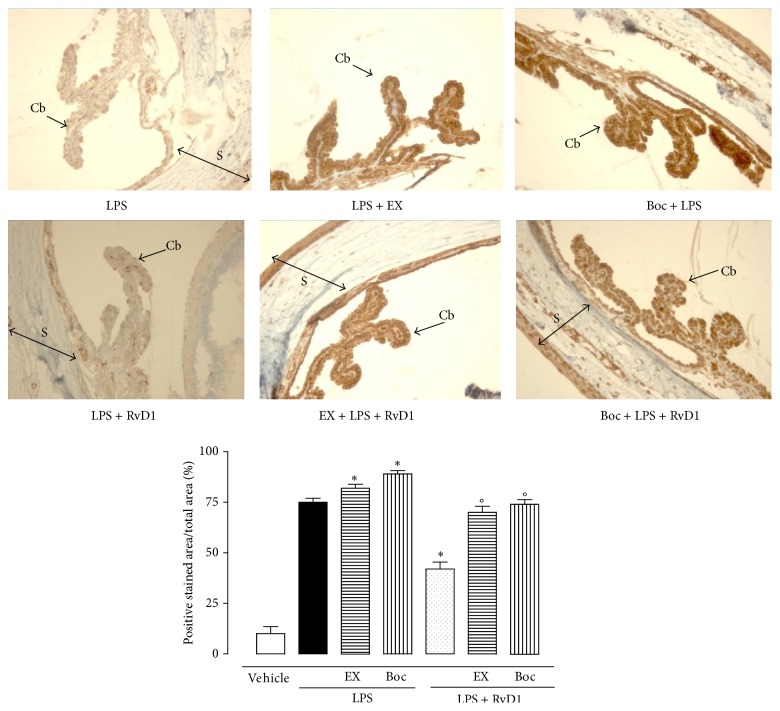
Representative immunohistochemistries of ocular tissues showing FOXO1 following LPS alone; LPS + RvD1 (1000 ng/kg); EX-527 + LPS + RvD1 (1000 ng/kg); Boc2 + LPS + RvD1 (1000 ng/kg). As shown by the percentage of stained area per total area analyzed, RvD1 decreased FOXO1 expression. The addition of EX-527 and Boc2 increases the expression of FOXO1 with respect to LPS alone, while they decrease the expression of this factor if administered prior to LPS + RvD1 (1000 ng/kg). Values are mean ± S.E.M. of *n* = 6 observations for each group. ^*∗*^ *P* < 0.05 and ^*∗∗*^ *P* < 0.01 versus LPS-treated group, while differences versus LPS + RvD1 1000 ng/kg are indicated as °*P* < 0.01. CB = ciliary body; S = sclera; EX = EX527; Boc = Boc2; RvD1 = resolvin D1.

Intravitreal RvD1 (10, 100, 1000 ng/kg) significantly increases the expression of SIRT1 into the eye as shown in Figure 2. This effect was significantly prevented by the treatment with RvD1 receptor antagonist Boc2 injected into the eyes before LPS and LPS + RvD1 (1000 ng/kg) (Figure 2). The increase in SIRT1 expression following RvD1 was accompanied by decrease of p53 and increase of FOXO1, starting from the lowest dose of RvD1 10 ng/kg (Figures 3(a) and 3(b)) as shown by western blotting of tissue homogenates. This data was confirmed by the immunohistochemical examination. Indeed, Figures 4 and 5 show the changes induced by RvD1 on p53 and FOXO1 abolished by Boc2. On another note, EX-527 before LPS + RvD1 increased the percentage of the total positive stained area/total area analyzed for both p53 and FOXO1 (*P* < 0.01 versus LPS and *P* < 0.01 versus LPS + RvD1) (Figures 4 and 5).

### 3.3. Intravitreal RvD1 and SIRT1 Related miRNAs

**Figure 6:**
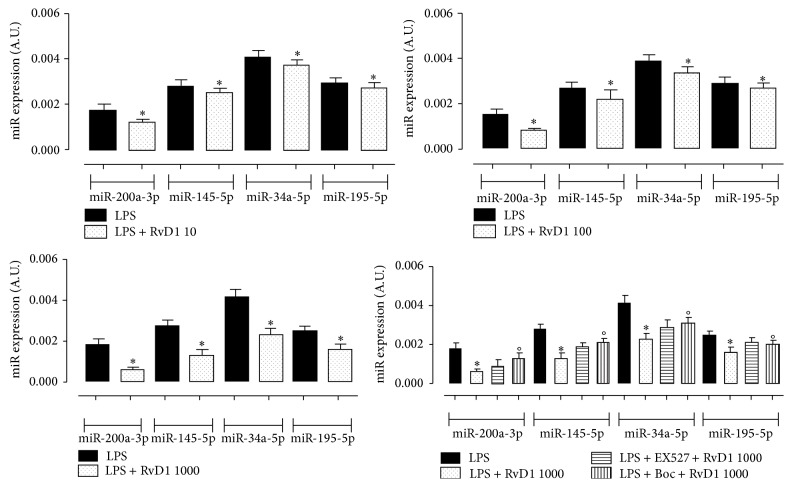
miRNAs expression (A.U., arbitrary units) in LPS-treated rats and LPS + RvD1 (10, 100, 1000 ng/kg) into the vitreous. Values are mean ± S.E.M. of *n* = 6 observations for each group. ^*∗*^ *P* < 0.05 and ^*∗∗*^ *P* < 0.01 versus LPS-treated group, while differences versus LPS + RvD1 1000 ng/kg groups are shown as °*P* < 0.01. Boc = Boc2; RvD1 = resolvin D1.

Comparing the miRNAs expression of LPS-treated rats with LPS + RvD1, a significant downregulation of the levels of miR-195-5p, miR-200a-3p (related to SIRT1 expression and activity), miR-145-5p (related to both SIRT1 and p53 regulation), and miR-34a-5p (candidate for p53 regulation) in ocular tissues of LPS + RvD1 1000 ng/kg treated rats compared to the LPS-treated group (*P* < 0.01 versus LPS group; Figure 6) was noted. These changes were abolished by Boc2 prior to RvD1 1000 ng/kg (Figure 6).

### 3.4. Caspase 3 and MnSOD

**Figure 7:**
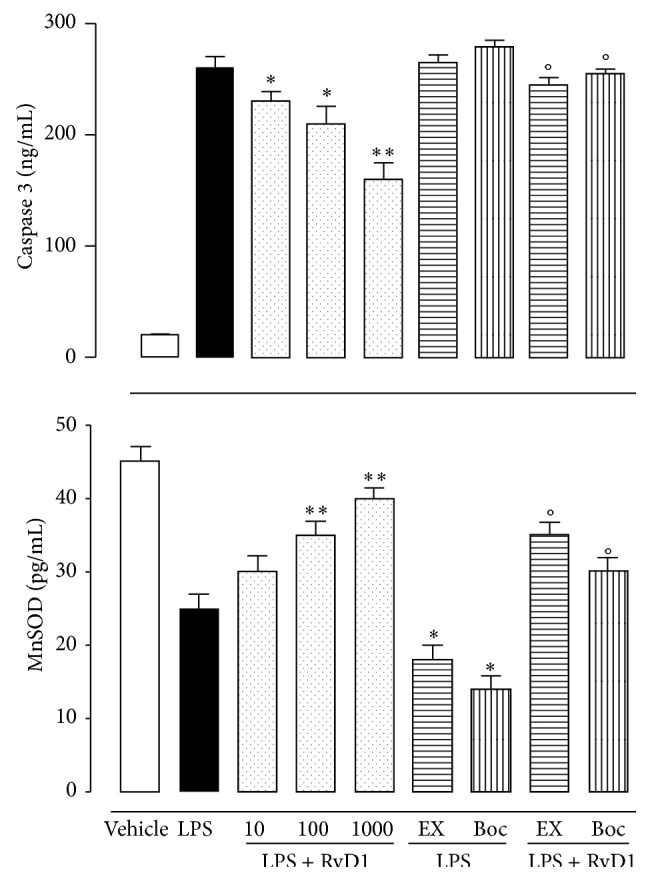
MnSOD and caspase 3 levels in ocular tissues of LPS-treated rats after RvD1 (10, 100, 1000 ng/kg), EX-527, and Boc2. Results are the mean ± S.E.M. of 6 observations per group. Significant differences with the LPS are shown as ^*∗*^ *P* < 0.05 and ^*∗∗*^ *P* < 0.01, while differences versus RvD1 1000 ng/kg are shown as °*P* < 0.01. EX = EX527; Boc = Boc2; RvD1 = resolvin D1.

Following intravitreal RvD1 in EIU rats, the levels of caspase 3 within the eye homogenates were diminished (Figure 7). In contrast, MnSOD was increased. The average of the RvD1 effect was 28%. To investigate the effects of the pharmacological modulation of SIRT1 on caspase 3 and MnSOD, Ex-527 and Boc2 treatment were performed as previously described prior to intravitreal injection of LPS + RvD1 (1000 ng/kg). Ex527 prior to LPS + RvD1 1000 ng/kg significantly reported these two markers MnSOD and caspase 3 towards the values expressed following the treatment with LPS alone (Figure 7).

## 4. Discussion

Previous work in our laboratory has shown that intravitreal RvD1 has a proresolutive role against endotoxic induced uveitis in rats, via modulation of the immune-inflammatory process in the eye [12]. In addition to this first evidence, the present study shows that, blocking the activity of the endogenous SIRT1 with the specific inhibitor EX527 [21], the protective effect of the intravitreal RvD1 diminished. Also, blocking the receptor for RvD1 FPR2/ALX with the specific antagonist Boc2, a diminution of the local levels of the endogenous SIRT1 was observed. Our results underline that RvD1 in addition to activating its own signal transduction pathway also promotes mobilization of the endogenous SIRT1 and thus activation of its specific pathway. In light of this and with Boc2 being a specific RvD1 receptor antagonist and EX527 being an inhibitor of SIRT activity, we were expected to observe that the RvD1 antagonist completely blocked the protection of RvD1 and the EX527 partly blocked it.

Interestingly, the protective action of intravitreal RvD1 was associated with an increased level of endogenous SIRT1 within the eye and a downregulated expression of miR-195-5p, miR-200a-3p, miR-34a-5p, and miR-145-5p. These miRNAs are notoriously and inversely related to SIRT1 expression in tissues as, for example, 195-5p and miR-200a-3p relate to SIRT1 expression and activity, miR-145-5p relates to SIRT1 and p53 levels, and miR-34a-5p relates to p53 regulation [9–11]. Because of this, one would argue that a diminished activity of SIRT1 causes diminished eye protection from RvD1 through changes of miRNAs.

miRNAs are a class of endogenous noncoding, single-stranded RNA (about 21–25 nucleotides) that are involved in the modulation of gene expression at posttranscriptional level in various biological processes including inflammation and oxidative stress [23, 24] by targeting mRNAs for degradation or by inhibiting translation [25]. mir-200a, mir-34, mir-195, and mir-381-3p are usually downregulated in presence of SIRT1 expression, and vice versa low expression of SIRT1 relates to miRNAs upregulation [9, 11, 17, 18, 26–28]. Also, upregulation of mir-34c-5p and mir-381-3p that target Nampt, an enzyme involved in the production of NAD, the cofactor for SIRT1 [29], diminishes SIRT1 actions. Noteworthy, each one of these miRNAs regulates several mRNAs which cooperate with several responses and in our case the final results of this cooperation seem to be the regulation of the immune-inflammatory response within the eye of uveitic rats.

SIRT1 belongs to a family of proteins called SIRT 1–7 with pathophysiological roles not completely explored. SIRT1 is a NAD^+^-dependent protein that regulates transcriptional activities via silencing of chromatin by histone deacetylation [30]. SIRT1 is ubiquitously expressed within different structures [31, 32], and it is not new that it is involved in several ocular pathologies including uveitis [4–8, 33–35] where SIRT1 exerts eye protection through reduction of cellular and molecular inflammatory responses, LPS-induced oxidative damage, and redox-sensitive NF-*κ*B deactivation [6]. However, to our knowledge this is the first time that a partial mediation by SIRT1 on the RvD1 eye protection has been depicted. This effect was also confirmed by the analysis of one of the factors regulated by SIRT1 in its expression, the acetyl-p53 and acetyl-FOXO1. Our western blotting showed a decreased expression of acetylated p53 and no increase of acetyl-FOXO1 in uveitic tissues that occurred after the stimulation of intravitreal RvD1 and blockade of the SIRT1 activity with EX527. p53 and FOXO are some of the numerous no-histone proteins targeted by SIRT1 together with such NF-*κ*B and mKu70 that are involved in DNA repair and factors involved in the nitric oxide synthase [2, 3, 30, 36]. Together with SIRT1 they orchestrate the mechanisms of the induction/resolution of inflammation. On another note, we observed a substantial diminution of the activity of RvD1 on MnSOD and caspase 3 in uveitic eye after blockade of the activity of SIRT1. This latter evidence is in accordance with the concept that oxidative stress negatively influences SIRT1 expression and activity [9, 11, 37–40], and vice versa activated SIRT1 increases cellular resistance to oxidative stress and apoptosis [41–44]. SIRT1 by regulating the expression of antioxidant agent such as MnSOD [45] also increases the endogenous defenses against oxidative stress.

In conclusion, the present study identifies RvD1 and SIRT1 as positive cooperators in the resolution of EIU. All together these results indicate interplay between intravitreal RvD1 and local SIRT1 in the protection from EIU, with SIRT1 that partly mediates the RvD1 protection. How this would happen warrants further and deeper investigation.

## References

[1] Mérida S., Sancho-Tello M., Navea A., Almansa I., Muriach M., Bosch-Morell F. (2014). An anti-interleukin-2 receptor drug attenuates T-helper 1 lymphocytes-mediated inflammation in an acute model of endotoxin-induced uveitis. *PLoS ONE*.

[2] Michan S., Sinclair D. (2007). Sirtuins in mammals: insights into their biological function. *Biochemical Journal*.

[3] Imai S.-I., Guarente L. (2010). Ten years of NAD-dependent SIR2 family deacetylases: implications for metabolic diseases. *Trends in Pharmacological Sciences*.

[4] Shindler K. S., Ventura E., Rex T. S., Elliott P., Rostami A. (2007). SIRT1 activation confers neuroprotection in experimental optic neuritis. *Investigative Ophthalmology and Visual Science*.

[5] Anekonda T. S., Adamus G. (2008). Resveratrol prevents antibody-induced apoptotic death of retinal cells through upregulation of Sirt1 and Ku70. *BMC Research Notes*.

[6] Kubota S., Kurihara T., Mochimar H. (2009). Prevention of ocular inflammation in endotoxin-induced uveitis with resveratrol by inhibiting oxidative damage and nuclear factor-*κ*B activation. *Investigative Ophthalmology and Visual Science*.

[7] Peng C.-H., Chang Y.-L., Kao C.-L. (2010). SirT1-a sensor for monitoring self-renewal and aging process in retinal stem cells. *Sensors*.

[8] Peng C.-H., Cherng J.-Y., Chiou G.-Y. (2011). Delivery of Oct4 and SirT1 with cationic polyurethanes-short branch PEI to aged retinal pigment epithelium. *Biomaterials*.

[9] Yamakuchi M., Ferlito M., Lowenstein C. J. (2008). miR-34a repression of SIRT1 regulates apoptosis. *Proceedings of the National Academy of Sciences of the United States of America*.

[10] Chen J.-H., Jones R. H., Tarry-Adkins J., Smith N. H., Ozanne S. E. (2008). Adverse effects of reduced oxygen tension on the proliferative capacity of rat kidney and insulin-secreting cell lines involve DNA damage and stress responses. *Experimental Cell Research*.

[11] Eades G., Yao Y., Yang M., Zhang Y., Chumsri S., Zhou Q. (2011). miR-200a regulates SIRT1 expression and epithelial to mesenchymal transition (EMT)-like transformation in mammary epithelial cells. *Journal of Biological Chemistry*.

[12] Rossi S., Di Filippo C., Ferraraccio F., Simonelli S., D'Amico M. (2012). Resolvin D1 reduces the immunoinflammatory response of the rat eye following uveitis. *Mediators of Inflammation*.

[13] Franks W. A., Limb G. A., Stanford M. R. (1992). Cytokines in human intraocular inflammation. *Current Eye Research*.

[14] Demir T., Gödekmerdan A., Balbaba M., Türkçüoglu P., Ilhan F., Demir N. (2006). The effect of infliximab, cyclosporine A and recombinant IL-10 on vitreous cytokine levels in experimental autoimmune uveitis. *Indian Journal of Ophthalmology*.

[15] Hosseini S. H., Salehifar E. (2009). Social phobia following maprotiline: the first case report. *Cases Journal*.

[16] Thrimawithana T. R., Young S., Bunt C. R., Green C., Alany R. G. (2011). Drug delivery to the posterior segment of the eye. *Drug Discovery Today*.

[17] Chen F., Xie Z., Wu X. (2012). Intravitreal injection of soluble erythropoietin receptor exacerbates photoreceptor cell apoptosis in a rat model of retinal detachment. *Current Eye Research*.

[18] Chen F., Hu S.-J. (2012). Effect of microRNA-34a in cell cycle, differentiation, and apoptosis: a review. *Journal of Biochemical and Molecular Toxicology*.

[19] Tian L.-L., Ren B., Gao X.-W. (2014). Inhibition of retinopathy of prematurity in rat by intravitreal injection of sorafenib. *International Journal of Ophthalmology*.

[20] Romano M. R., Biagioni F., Besozzi G. (2012). Effects of bevacizumab on neuronal viability of retinal ganglion cells in rats. *Brain Research*.

[21] Kim D. H., Jung Y. J., Lee J. E. (2011). Sirt1 activation by resveratrol ameliorates cisplatin-induced renal injury through deacetylation of p53. *The American Journal of Physiology—Renal Physiology*.

[22] Gavins F. N. E., Kamal A. M., D'Amico M., Oliani S. M., Perretti M. (2005). Formyl-peptide receptor is not involved in the protection afforded by annexin 1 in murine acute myocardial infarct. *The FASEB Journal*.

[23] di Francesco A., de Pittà C., Moret F., Barbieri V., Celotti L., Mognato M. (2013). The DNA-damage response to *γ*-radiation is affected by miR-27a in A549 cells. *International Journal of Molecular Sciences*.

[24] Bae S., Lee E.-J., Lee J. H. (2014). Oridonin protects HaCaT keratinocytes against hydrogen peroxide-induced oxidative stress by altering microRNA expression. *International Journal of Molecular Medicine*.

[25] Loscher C. J., Hokamp K., Wilson J. H. (2008). A common microRNA signature in mouse models of retinal degeneration. *Experimental Eye Research*.

[26] Luan S., Sun L., Huang F. (2010). MicroRNA-34a: a novel tumor suppressor in p53-mutant glioma cell line U251. *Archives of Medical Research*.

[27] Zhu H., Yang Y., Wang Y., Li J., Schiller P. W., Peng T. (2011). MicroRNA-195 promotes palmitate-induced apoptosis in cardiomyocytes by down-regulating Sirt1. *Cardiovascular Research*.

[28] Mortuza R., Feng B., Chakrabarti S. (2014). miR-195 regulates SIRT1-mediated changes in diabetic retinopathy. *Diabetologia*.

[29] Imai S.-I. (2009). Nicotinamide phosphoribosyltransferase (Nampt): a link between NAD biology, metabolism, and diseases. *Current Pharmaceutical Design*.

[30] Tegla C. A., Azimzadeh P., Andrian-Albescu M. (2014). SIRT1 is decreased during relapses in patients with multiple sclerosis. *Experimental and Molecular Pathology*.

[31] Haigis M. C., Guarente L. P. (2006). Mammalian sirtuins—emerging roles in physiology, aging, and calorie restriction. *Genes & Development*.

[32] Gertz M., Steegborn C. (2010). Function and regulation of the mitochondrial Sirtuin isoform Sirt5 in Mammalia. *Biochimica et Biophysica Acta*.

[33] Jaliffa C., Ameqrane I., Dansault A. (2009). Sirt1 involvement in rd10 mouse retinal degeneration. *Investigative Ophthalmology & Visual Science*.

[34] Ozawa Y., Kubota S., Narimatsu T. (2010). Retinal aging and sirtuins. *Ophthalmic Research*.

[35] Geng Y. Q., Li T. T., Liu X. Y., Li Z. H., Fu Y. C. (2011). SIRT1 and SIRT5 activity expression and behavioral responses to calorie restriction. *Journal of Cellular Biochemistry*.

[36] Buck S. W., Gallo C. M., Smith J. S. (2004). Diversity in the Sir2 family of protein deacetylases. *Journal of Leukocyte Biology*.

[37] Cohen H. Y., Miller C., Bitterman K. J. (2004). Calorie restriction promotes mammalian cell survival by inducing the SIRT1 deacetylase. *Science*.

[38] Brunet A., Sweeney L. B., Sturgill J. F. (2004). Stress-dependent regulation of FOXO transcription factors by the SIRT1 deacetylase. *Science*.

[39] Liu D., Gharavi R., Pitta M., Gleichmann M., Mattson M. P. (2009). Nicotinamide prevents NAD^+^ depletion and protects neurons against excitotoxicity and cerebral Ischemia: NAD^+^ consumption by sirt1 may endanger energetically compromised neurons. *NeuroMolecular Medicine*.

[40] Yuan J., Luo K., Liu T., Lou Z. (2012). Regulation of SIRT1 activity by genotoxic stress. *Genes & Development*.

[41] He W., Wang Y., Zhang M.-Z. (2010). Sirt1 activation protects the mouse renal medulla from oxidative injury. *The Journal of Clinical Investigation*.

[42] Chen Z., Peng I.-C., Cui X., Li Y.-S., Chien S., Shyy J. Y.-J. (2010). Shear stress, SIRT1, and vascular homeostasis. *Proceedings of the National Academy of Sciences of the United States of America*.

[43] Yan W., Fang Z., Yang Q. (2013). SirT1 mediates hyperbaric oxygen preconditioning-induced ischemic tolerance in rat brain. *Journal of Cerebral Blood Flow and Metabolism*.

[44] Wu D., Hu Q., Liu X., Pan L., Xiong Q., Zhu Y. Z. (2015). Hydrogen sulfide protects against apoptosis under oxidative stress through SIRT1 pathway in H9c2 cardiomyocytes. *Nitric Oxide*.

[45] Alcendor R. R., Gao S., Zhai P. (2007). Sirt1 regulates aging and resistance to oxidative stress in the heart. *Circulation Research*.

